# EduSCARA: An open source RRPR educational SCARA platform

**DOI:** 10.1016/j.ohx.2026.e00761

**Published:** 2026-03-17

**Authors:** Alex Clark, Uriel Martinez-Hernandez, Tareq Assaf

**Affiliations:** Department of Electronic & Electrical Engineering, University of Bath, Bath, UK

**Keywords:** Educational, Small robot, SCARA, Closed-loop, 3D printed, Open source

## Abstract

This paper presents EduSCARA, an affordable and industrially relevant Selective Compliance Assembly Robot Arm (SCARA) development platform. Designed to lower the barrier to industrial robotics, EduSCARA provides a complete, hands-on environment that closely replicates the architecture and challenges of professional systems without the prohibitive cost or complexity. The manipulator features a 3D-printed RRPR design actuated by hobby-grade servomotors, with PID-tunable closed-loop control on the planar axes. Its modular and transparent design supports both hardware and firmware customisation, while the Python API gives learners a versatile platform for experimentation. Validation of the system shows ± 3.5 mm repeatability at high speeds and a 100 g payload capacity, supporting small-scale educational and practical tasks. In contrast to traditional industrial robots, often costing tens of thousands of pounds, EduSCARA empowers learners to experiment, debug, and innovate at a fraction of the cost of many other educational robotics platforms. More than just a small robot, EduSCARA serves as a gateway to industrial robotics, making high-impact, professional-grade learning truly accessible.

## Graphical abstract

**Figure:**
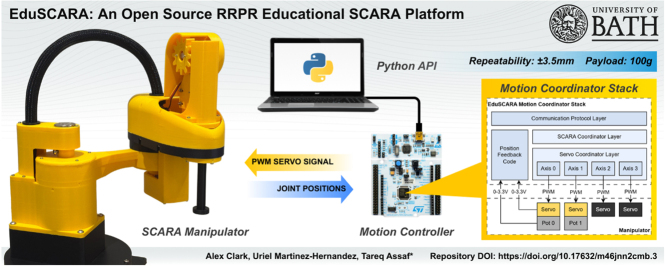

## Specifications table

**Table:** 

Hardware name	*EduSCARA*
Subject area	•*Educational tools and open source alternatives to existing infrastructure*

Hardware type	•*Electrical engineering and computer science* •*Robotics engineering*

Closest commercial analog	Closest commercial analog: Igus SCARA robots for laboratories. Differences: An affordable, 3D-printable RRPR-type SCARA with closed-loop control on axes 0 and 1, designed for teaching; uses widely available components and is open source.

Open source license	CC BY 4.0

Cost of hardware	£111.50

Source file repository	https://doi.org/10.17632/m46jnn2cmb.3

## 1 Hardware in context

Practical robotics education bridges the gap between theoretical knowledge and real-world application, effectively deepening understanding of autonomous systems [1]. It exposes students to the non-idealities, system limitations and debugging challenges that are fundamental to developing successful robotic systems. EduSCARA provides a realistic and accessible environment for students to experiment, prototype, and develop real-world applications using a platform that closely mirrors industrial systems. This initiative aligns with the framework for robotic roles in education proposed by Xu and Ouyang, specifically targeting ‘Learning through robotics’ [2].

This approach positions educational robotics as tools or assistants, enabling students to actively engage in the educational process by utilising robots to achieve specific objectives. The platform is designed to offer a low-risk, hands-on learning experience suitable for educational environments. Prior research supports the effectiveness of such approaches in improving problem-solving, computational thinking, and self-efficacy [3]. By creating a learning environment that avoids the high costs and risks associated with industrial robotics, the transition to real-world robotics engineering roles becomes less daunting and more accessible.

Industrial robots are available in several common configurations, including Articulated, SCARA, Cartesian/gantry, Parallel (Delta), and Collaborative designs. These robots are widely used across industrial automation, each offering different mechanical characteristics and suitability for specific tasks. Articulated robots dominate global industrial deployment, whereas collaborative robots have seen strong and accelerating growth [4], [5], [6].

This general distribution helps explain why the educational robotics market is saturated with articulated robotic arms, such as those offered by Niryo [7]. Cartesian/gantry systems also have a strong presence in educational environments, particularly through their use in 3D printers, CNC machines and laser cutters. Despite SCARA robots being prevalent in industrial applications they are notably underrepresented in education settings. EduSCARA addresses this imbalance by providing a SCARA-based solution tailored to educational use.

SCARA robots are highly relevant in industrial automation, commonly used in applications such as pick-and-place, assembly, inspection, and packaging [8], offering high speed and precise positioning capabilities [9]. Available options for practical robotics education are industrial robot systems and educational robot platforms.

Industrial SCARA robots from manufacturers such as ABB and KUKA offer the highest levels of fidelity and realism. However, they come at a significant cost, typically tens of thousands of pounds per unit. Additional installation and maintenance expenses contribute significantly to the total system cost. Moreover, their use requires specialised training, dedicated safety systems, and appropriate infrastructure. This makes them impractical for hands-on education at scale, particularly for early-stage learners who face a higher risk of injury or damaging equipment. These high stakes discourage experimentation and trial-and-error learning, which are both essential for developing practical robotics skills. This highlights the need for dedicated educational robotic platforms that can overcome these limitations in academic settings.

A well-known example of an educational SCARA platform is the Arduino-based SCARA robot developed by *How To Mechatronics* [10]. Several other designs, such as the 5-DOF SCARA robot arm developed at Prasetiya Mulya University [11], adopt an *RPRR* (Revolute-Prismatic-Revolute-Revolute) configuration, where the vertical (Z-axis) motion is handled by a linear actuator mounted at the base. The main advantages are its straightforward mechanical design and the reduced load on the moving arm, as it does not need to carry the weight of the Z-axis actuator or its associated mechanism [12].

**Fig. 1:**
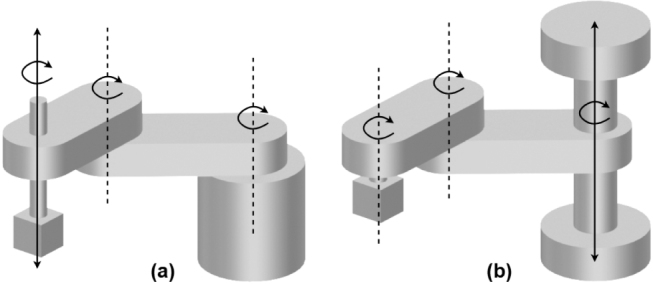
RRPR (a) and RPRR (b) SCARA configurations.

In contrast, most industrial SCARA robots utilise an *RRPR* (Revolute-Revolute-Prismatic-Revolute) configuration [13], in which the Z-axis actuator is mounted in line with the wrist. RRPR SCARA manipulators are advantageous in light-duty applications that require precision and speed [14] – characteristics that closely align with the typical industrial use cases of SCARA robots, which explains their widespread adoption. An example of an educational SCARA with this configuration is the ‘ROBCO’ SCARA [15]. A comparison between RRPR and RPRR SCARA configurations is illustrated in Fig. 1.

To maximise its educational value, the EduSCARA manipulator adopts the RRPR configuration, widely used in industry yet still underrepresented in educational settings. Unlike previous open-loop examples, it features closed-loop control on key axes, allowing students to engage with critical aspects of industrial robotics such as control system tuning. Table 1 presents a feature comparison between the EduSCARA platform, the educational robotic systems mentioned previously, and educational robotic arms currently published on HardwareX.

The EduSCARA platform provides not only a compact manipulator but also the supporting system required for realistic robotics application development. The arm is actuated directly by four hobby-grade servomotors, avoiding belt transmissions, resulting in a mechanically rigid structure. Axes 0 and 1 use potentiometer feedback for closed-loop control, while axes 2 and 3 operate open-loop. Feedback is limited to the planar joints to reduce weight, cost, and complexity while still enabling effective hands-on learning with closed-loop control. The Motion Controller firmware implements the servo control system, forward and inverse kinematics, and double-S motion profiling for joints moves. The Python API provides a high-level interface well suited for education and increasingly aligned with industry workflows involving computer vision and AI. These elements are described in detail in the Hardware Description section.

**Table 1:** Feature comparison of the EduSCARA and related educational robots.

Robot name	Configuration	Key features	Payload	Repeatability	Reach	Cost
Niryo Ned 2 [7]	6-DOF Articulated	Stepper motors + encoders, Niryo Studio,	300 g	0.5 mm	490 mm	£4460
		Python, ROS, Modbus, Matlab API				

Prasetiya Mulya	RPRR SCARA	Stepper motors, Jogging GUI	–	14 mm	–	–
University SCARA [11]						

ROBCO SCARA [15]	RRPR SCARA	Stepper motors, Programming GUI + CLI	–	–	–	–

IoT robot arm	2-DOF Jointed-arm	Hobby servos, Smartphone app	–	–	190.8 mm	£22
(HardwareX) [16]						

PARA robotic arm	3-DOF Jointed-arm	Industrial servos, URDF file	2 kg	2.6 mm	940 mm	£2548
(HardwareX) [17]						

EduSCARA	RRPR SCARA	Hobby servos + potentiometers (axis 0, 1),	100 g	3.5 mm	225 mm	£112
		Motion controller, Python API				

## 2 Hardware description

**Fig. 2:**
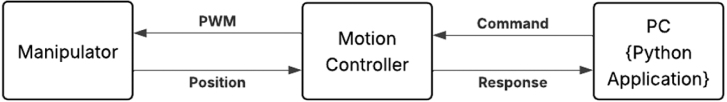
High-level block diagram of the EduSCARA platform.

The EduSCARA platform consists of a 3D-printable RRPR SCARA Manipulator and a Motion Controller, forming a closed-loop SCARA system as illustrated in Fig. 2. This section presents a detailed description of the system architecture, beginning with the actuation approach and mechanical structure of the manipulator, followed by an overview of the electrical and signal architecture. The Motion Controller hardware and firmware are then discussed in detail, and the Python API is presented to provide a complete understanding of the platform’s design.

Servo motors and stepper motors, both of which are popular in small-scale robotic systems, offer distinct advantages and trade-offs. For the EduSCARA manipulator, servo motors were selected as the preferred actuators due to several advantages that align well with the project’s educational and structural goals. Some key factors include their ease of use, compact size, low weight, compatibility with low-voltage DC power supplies and notably, their lower operating temperatures. Temperature is a critical factor, as stepper motors can reach 100 °C (ambient 20 °C) [18] during operation due to constant current draw [19]. This poses a risk when using 3D-printed PLA [20] or PETG [21] (Bambu Lab) components, as both materials have heat deflection temperatures below this limit. PLA was selected over PETG because it offers superior mechanical properties at room temperature.

Potentiometers were selected as the position feedback sensors because they offer functionality similar to absolute encoders used in industrial SCARA systems, while being significantly more affordable and simpler to implement. Unlike incremental encoders, they do not require end-stops or homing sequences at startup. The potentiometer resistance tolerance specifies the allowable variation in total end-to-end resistance. Single-turn potentiometers with resistance tolerances below 5% were prohibitively expensive; therefore, a lower-cost device with a 10% tolerance was selected. Combined with adjustment travel and mechanical travel errors, this can introduce significant inaccuracies in joint angle measurements. To address this, these sources of error are mitigated through manual calibration of each potentiometer against the actual joint angle, rather than relying on nominal device parameters. This calibration procedure is described in the Potentiometer Calibration section.

**Fig. 3:**
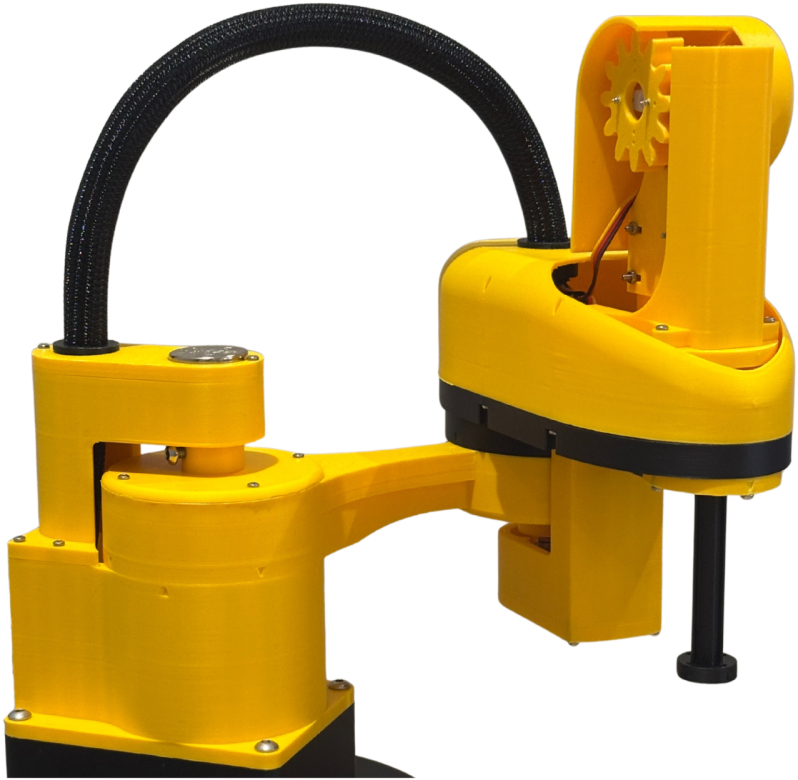
EduSCARA manipulator.

The EduSCARA manipulator is shown in Fig. 3. Its link lengths are fully customisable to suit different applications, with support for arbitrary dimensions easily configured through the API. The default link lengths are L1 = 125 mm and L2 = 100 mm. Extending the reach beyond these values is not recommended without reinforcing the links to accommodate the increased moment load. While performance is limited compared to industrial systems, it does replicate the core mechanical structure and control principles of a real SCARA, making it an ideal testbed for applying control theory and motion planning.

Unlike proprietary industrial motion controllers, the EduSCARA motion controller uses an STM32 microcontroller with open-source firmware. This provides full access to the EduSCARA Motion Coordinator Stack, supporting educational engagement with embedded programming for motion control. The controller integrates with a Python-based API, enabling straightforward interaction with external tools such as computer vision systems and machine learning frameworks.

The EduSCARA platform offers several ways in which it can be valuable to other researchers across both standard and novel laboratory contexts:

• An accessible testbed for manipulation research, allowing rapid prototyping of grippers, sensors, or task fixtures for pick-and-place, sorting, and small-object handling experiments.
• A low-cost platform for robotics education, enabling demonstrations of kinematics, calibration, motor control, and automation principles without the need for expensive industrial hardware.
• A customisable mechanical framework using 3D-printed components, allowing researchers to reuse or adapt the arm geometry, joints, or electronics for new robotic designs.
• A basis for developing small-scale applications, such as the Resistor Sorting task demonstrated later in Teaching Exercise Example – Resistor Sorter.

### 2.1 Mechanical system

**Fig. 4:**
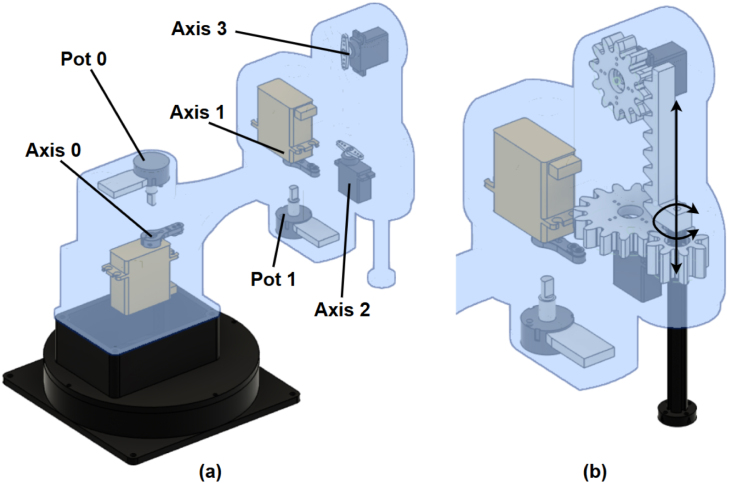
Axes 0, 1, 2 and 3 locations (a) and Z mechanism (b).

The EduSCARA manipulator implements an RRPR configuration, as illustrated in Fig. 4. Its base is weighted with a 1.25 kg mass, allowing it to stand freely, and features screw holes for secure mounting to the operating surface. The first joint is actuated by a servo motor (Axis 0) with a 180-degree range, and real-time position feedback provided by a single-turn potentiometer mounted directly above. The second joint, mounted at 90 degrees relative to the first, is actuated by a servo motor (Axis 1) with a single-turn potentiometer mounted beneath. This design maximises the working area given the limited servo range, though it restricts operation to an elbow-up (left-handed) configuration. Together, Axes 0 and 1 control the end-effector’s X and Y positioning. A servo motor (Axis 2) drives a 1:2 spur gear setup providing full 360-degree Z rotation of the end-effector, while Z positioning is handled via a servo-driven rack-and-pinion mechanism (Axis 3).

### 2.2 Electrical system

**Fig. 5:**
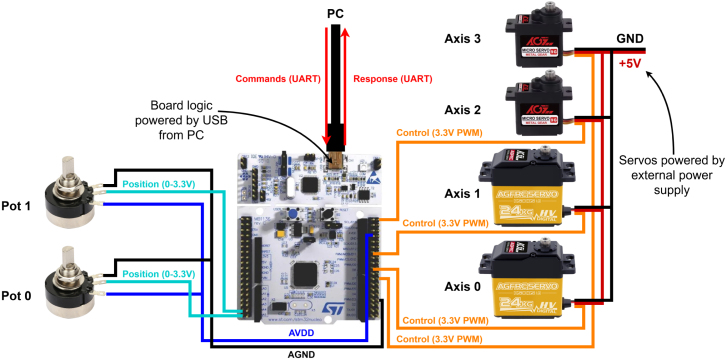
Electrical diagram of EduSCARA platform.

The electrical diagram of EduSCARA is shown in Fig. 5. The controller board logic, potentiometers, and servo PWM signals are powered through the board’s USB connection (5 V line reduced to 3.3 V by the on-board regulator U4 [22]). The servos are powered separately from an external 5 V supply. The selected servo motors operate reliably using a 3.3 V PWM control signal. The EduSCARA motion controller is built on the NUCLEO-F446RE development board [23], a cost-effective platform that offers sufficient processing resources and peripherals. The microcontroller controls four servo motors (Axes 0–3) using PWM signals and receives analog feedback from potentiometers on Axes 0 and 1. Communication with the user’s PC is handled via a serial connection through the USB port (UART) on the development board.

Axes 0 and 1 use AGFRC B53DHS servo motors [24], chosen for their high torque (24kgcm) and programmability, allowing adjustment of parameters such as travel range, neutral position, damping, and sensitivity. Axes 2 and 3 use AGFRC B11DLS servo motors [25], metal-geared alternatives to SG90 servos, which provide sufficient torque (2.8kgcm) while being small and lightweight. For position feedback for Axes 0 and 1, RV24YN20F B502 potentiometers [26] were selected for their form factor which integrates well with the manipulator design.

### 2.3 Firmware

**Fig. 6:**
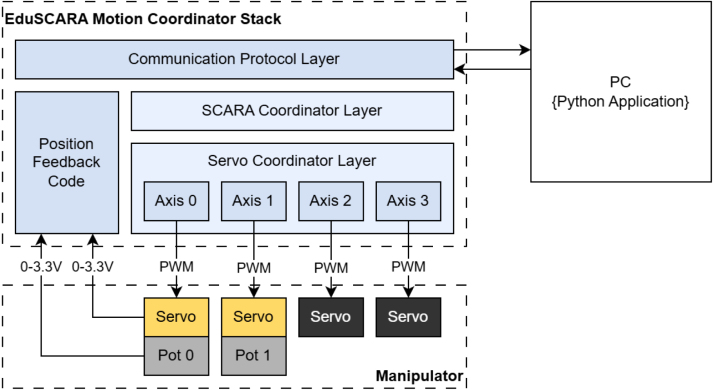
EduSCARA Motion Coordinator Stack.

The Motion Controller acts as an interface between the PC and the manipulator. The EduSCARA Motion Coordinator Stack, shown in Fig. 6, represents the firmware architecture implemented on the Motion Controller. The firmware is available in the repository, an STM32CubeIDE project named *servo_motion_controller*.

The stack is composed of several layers: the Communication Protocol Layer handles API commands from the PC, the SCARA Coordinator Layer manages forward and inverse kinematics, and the Servo Coordinator Layer generates velocity profiles and issues coordinated motion commands to individual Servo Instances, which produce the PWM signals for each motor. Additionally, the Position Feedback Code converts raw analog readings from the potentiometers into angular measurements in degrees and is referenced throughout the stack.

#### 2.3.1 Communication protocol layer (*application.c*)

**Fig. 7:**
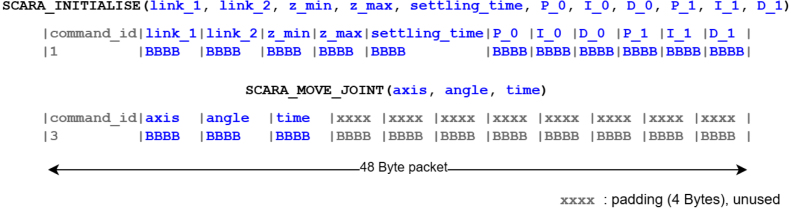
Packet structures of the SCARA_INITIALISE and SCARA_MOVE_JOINT API commands.

The Communication Protocol Layer handles commands transmitted over the UART2 serial interface (USB port) using a 48-byte (12-word) packet, as described in Fig. 7. Once a command is received, the command_id (first word) is used in a switch-case statement to determine the method for processing the received data. Upon successful command execution, a success_flag is transmitted back to the sender, signalling that it is ready to receive the next command.

#### 2.3.2 SCARA coordinator layer (*scara.c*)

**Fig. 8:**
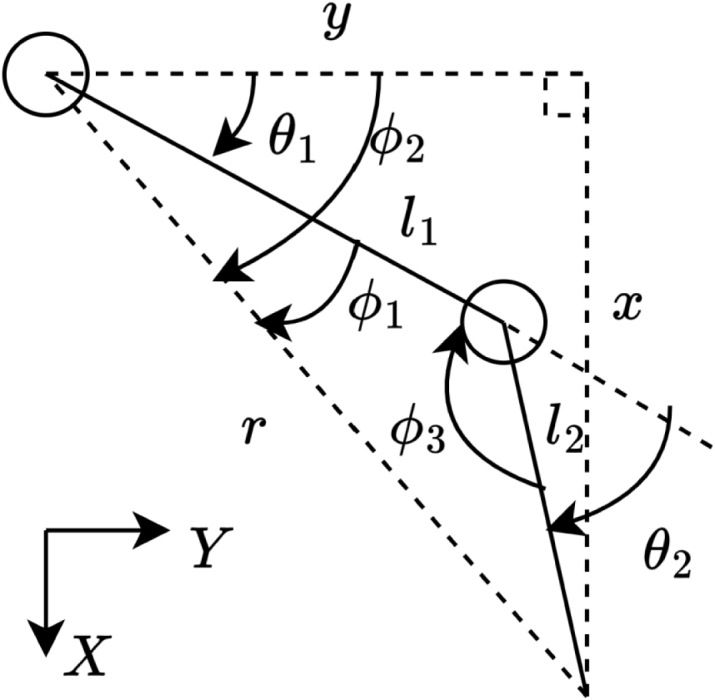
Left-handed SCARA in world frame.

The SCARA Coordinator Layer implements the forward and inverse kinematics for the manipulator configured for elbow-up (left-handed) mode. Fig. 8 defines the coordinate frame and variables referenced throughout the following kinematic equations for Axes 0 and 1.

The forward kinematics used to compute the end-effector position for the SCARA_READ_COORD command are defined by Eqs. (1), (2). Only the planar X and Y coordinates are calculated, as positional feedback is available exclusively on Axes 0 and 1. The reported Z position and end-effector rotation inferred directly from the PWM signals currently applied to Axes 2 and 3.

(1) $$x=l_{1}\sin\left(\theta_{1}\right)+l_{2}\sin\left(\theta_{1}+\theta_{2}\right)$$

(2) $$y=l_{1}\cos\left(\theta_{1}\right)+l_{2}\cos\left(\theta_{1}+\theta_{2}\right)$$

To determine the joint angles $\theta_{1}$ and $\theta_{2}$ required by the SCARA_MOVE_COORD command, the inverse kinematics are computed using the equations given below:

$$r=\sqrt{x^{2}+y^{2}}$$

$$\phi_{1}=\cos^{-1}\;\left(\frac{l_{2}^{2}-r^{2}-l_{1}^{2}}{-2rl_{1}}\right),\;\phi_{2}=\tan^{-1}\;\left(\frac{x}{y}\right),\;\phi_{3}=\cos^{-1}\;\left(\frac{r^{2}-l_{1}^{2}-l_{2}^{2}}{-2l_{1}l_{2}}\right)$$

(3) $$\theta_{1}=\phi_{2}-\phi_{1}$$

(4) $$\theta_{2}=180^{\circ }-\phi_{3}$$

**Fig. 9:**
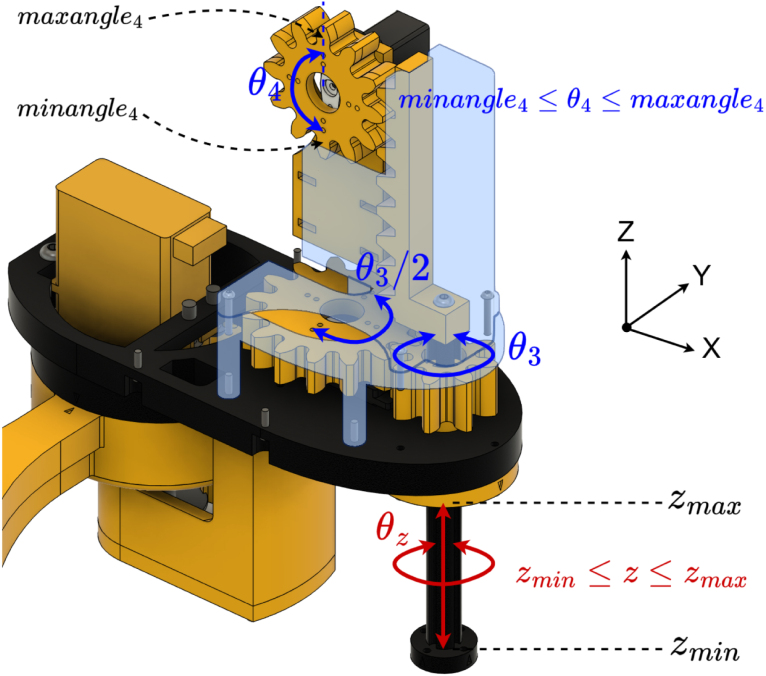
Z-axis mechanism in world frame.

The Z-axis mechanism is described in Fig. 9. The desired world–frame rotation $\theta_{Z}$ is achieved by rotating Axis 2 to $\theta_{3}$:

(5) $$\theta_{3}=\theta_{Z}-\left(\theta_{1}+\theta_{2}\right).$$

Axis 3 converts rotational motion into linear motion through a rack-and-pinion mechanism. The required angle of Axis 3 ($\theta_{4}$) to achieve the desired Z coordinate (z) is calculated by mapping the servo’s rotational range to the linear travel range of the Z-axis. The limits *z_min* and *z_max* are user-defined, as they depend on the end-effector attached to the Z-axis. $\theta_{4}$ is calculated using Eq. (6).

(6) $$\theta_{4}={\text{minangle}}_{4}+\left(\left(z-z\text{\_}min\right)\times \frac{{\text{maxangle}}_{4}-{\text{minangle}}_{4}}{z\text{\_}max-z\text{\_}min}\right)$$

#### 2.3.3 Servo coordinator layer (*servo_controller.c*)

**Fig. 10:**
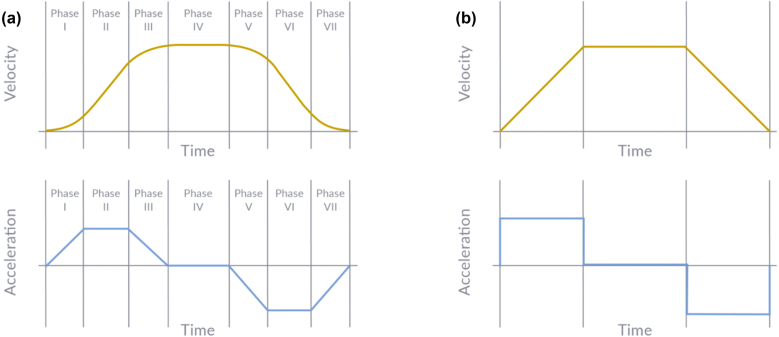
Double-S profile (a) and Trapezoidal profile (b) [27].

The servo coordinator layer uses a Double-S velocity profile, which smoothly ramps velocity to reduce settling time, mechanical stress, and vibration. A standard Trapezoidal profile consists of constant acceleration, constant velocity, and constant deceleration, but its instantaneous acceleration changes create infinite jerk at phase transitions. The Double-S profile overcomes this by introducing smooth, piecewise-linear acceleration with constant jerk across seven motion phases. A comparison with the Trapezoidal profile is shown in Fig. 10.

**Fig. 11:**
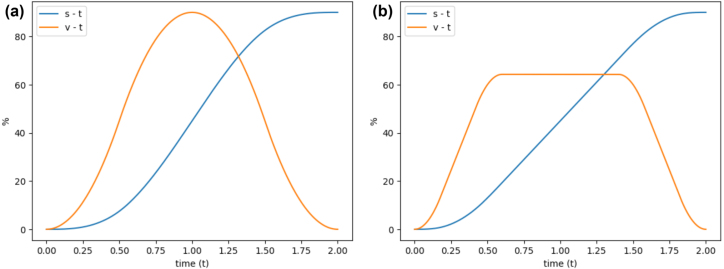
Resulting displacement (s-t) and velocity (v-t) profiles for (α=0.5,β=0.5) (a) and (α=0.3,β=0.3) (b).

EduSCARA move commands use the “Double-S trajectory with assigned phase durations” described in Section 3.2.6 of *Trajectory Planning for Automatic Machines and Robots* [28]. The phase durations are controlled by two parameters: α, which defines the proportion of total motion time spent accelerating and decelerating, and β, which defines the proportion of each acceleration/deceleration phase spent in the jerk segment. The servo coordinator layer uses default values of α=0.3 and β=0.3. Fig. 11 illustrates how adjusting these parameters shape the velocity profile.

**Fig. 12:**
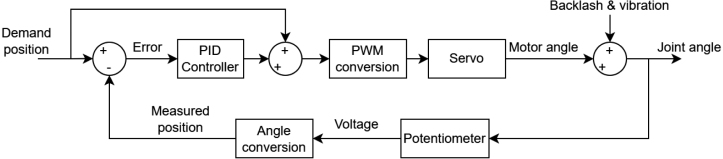
PID control system implementation for Axes 0 and 1.

If a move command involves Axis 0 or Axis 1, PID (Proportional–Integral–Derivative) control is applied throughout the motion. The P, I, and D gains are configured using the SCARA_INITIALISE command. Fig. 12 illustrates how PID control is implemented on these axes.

**Fig. 13:**
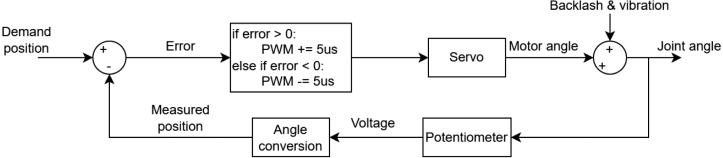
Optional post-move joint-angle correction control system implementation for Axes 0 and 1.

Backlash is the clearance between mating gears and is present in the hobby-grade servos used in EduSCARA. It contributes to lost motion, where movement at the output fails to match the commanded input. The selected servos also have a 4 microsecond deadband, meaning small input changes produce no motion, further reducing positioning accuracy. To compensate for these effects, a joint-angle correction routine can be applied after each completed movement, as shown in Fig. 13.

**Fig. 14:**
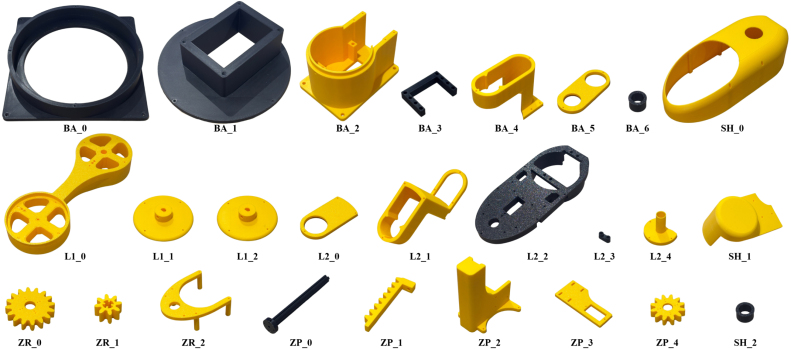
3D Printed Manipulator Components with Designators.

The correction control system adjusts the PWM by 5 microseconds each cycle and operates for a duration defined by settling_time, set using the SCARA_INITIALISE command. This feature can be disabled by setting settling_time to zero.

### 2.4 API

The EduSCARA Python API enables communication with the motion controller over a USB serial connection using the UART protocol, interfacing with the Communication Protocol Layer. The API is contained in a single Python file, *scara_motion_controller_api.py*, which can be used by placing it in the working directory and importing it into the application script. Descriptions of each API command are provided in Table 5.

## 3 Design files summary

The design filenames and their location in the Mendeley Data repository are shown in Table 2. Images of the 3D-printed components are shown in Fig. 14, and the descriptions of each component are listed below. All components were 3D printed in PLA on a BambuLab A1 using a 0.4 mm nozzle and a Textured PEI build plate. The slicing software used was BambuStudio. The majority of components can be printed without supports, however, some models (BA_4, SH_0, L1_0, L2_1, SH_1 and ZP_2) required ‘tree’ supports. The slicer configuration was based on the default BambuStudio settings for the A1 printer, with the following modifications: ‘Order of walls’ set to ‘inner wall/outer wall/inner wall’, ‘Enable support’ with type ‘tree(auto)’, and ‘Brim type’ set to ‘Outer brim only’.

**Table 2:** 3D Printable manipulator design files.

Designator	Design filename	File type	Open source licence	Location of the file
BA_0	base_0.step	CAD file	CC BY 4.0	base_0.step
BA_1	base_1.step	CAD file	CC BY 4.0	base_1.step
BA_2	base_2.step	CAD file	CC BY 4.0	base_2.step
BA_3	base_3.step	CAD file	CC BY 4.0	base_3.step
BA_4	base_4.step	CAD file	CC BY 4.0	base_4.step
BA_5	base_5.step	CAD file	CC BY 4.0	base_5.step
BA_6	base_6.step	CAD file	CC BY 4.0	base_6.step

L1_0	link1_0.step	CAD file	CC BY 4.0	link1_0.step
L1_1	link1_1.step	CAD file	CC BY 4.0	link1_1.step
L1_2	link1_2.step	CAD file	CC BY 4.0	link1_2.step

L2_0	link2_0.step	CAD file	CC BY 4.0	link2_0.step
L2_1	link2_1.step	CAD file	CC BY 4.0	link2_1.step
L2_2	link2_2.step	CAD file	CC BY 4.0	link2_2.step
L2_3	link2_3.step	CAD file	CC BY 4.0	link2_3.step
L2_4	link2_4.step	CAD file	CC BY 4.0	link2_4.step

ZR_0	zrot_0.step	CAD file	CC BY 4.0	zrot_0.step
ZR_1	zrot_1.step	CAD file	CC BY 4.0	zrot_1.step
ZR_2	zrot_2.step	CAD file	CC BY 4.0	zrot_2.step

ZP_0	zpos_0.step	CAD file	CC BY 4.0	zpos_0.step
ZP_1	zpos_1.step	CAD file	CC BY 4.0	zpos_1.step
ZP_2	zpos_2.step	CAD file	CC BY 4.0	zpos_2.step
ZP_3	zpos_3.step	CAD file	CC BY 4.0	zpos_3.step
ZP_4	zpos_4.step	CAD file	CC BY 4.0	zpos_4.step

SH_0	shell_0.step	CAD file	CC BY 4.0	shell_0.step
SH_1	shell_1.step	CAD file	CC BY 4.0	shell_1.step
SH_2	shell_2.step	CAD file	CC BY 4.0	shell_2.step

ASSEMBLY	EduSCARA.f3d	CAD assembly	CC BY 4.0	EduSCARA.f3d

1. **Weight Housing (BA_0)** - Contains the 1.25 kg weight, with screw holes to fix to a surface.
2. **Weight Lid (BA_1)** - Lid for BA_0, secures the robot to the weight. The Z-offset can be adjusted by altering the height of this component.
3. **Robot Base (BA_2)** - The base of the robot.
4. **Base Bracket (BA_3)** - Secures the servo motor (Axis 0) to the base.
5. **Pot 0 Housing (BA_4)** - Holds the Potentiometer directly above Axis 0.
6. **Pot 0 Lid (BA_5)** - Encloses Potentiometer and its wires, with a hole to feed the cable sleeve through.
7. **Cable Sleeve Bracket (BA_6)** - Inserted into BA_5 (interference fit). Supports the cable sleeve.
8. **Link 1 (L1_0)** - Link 1 of the Robot.
9. **Pot 0 Shaft Bracket (L1_1)** - Fixes the shaft of Pot 0 to Axis 0.
10. **Pot 1 Shaft Bracket (L1_2)** - Fixes the shaft of Pot 1 to Axis 1.
11. **Pot 1 Lid (L2_0)** - Encloses the Potentiometer and its wires.
12. **Pot 1 Housing (L2_1)** - Holds the Potentiometer directly below Axis 1.
13. **Link 2 (L2_2)** - Link 2 of the Robot.
14. **Link 2 Bracket (L2_3)** - Secures the servo motor (Axis 1) to Link 2.
15. **Z Gear Mount (L2_4)** - The mounting point for ZR_1, with ZR_0 held in position by the hollow shaft.
16. **Z Rotation Motor Gear (ZR_0)** - Mounted to servo motor (Axis 2). Connected to ZR_1 with 1:2 ratio, allowing for 360 degrees rotation of the Z-axis.
17. **Z Rotation Gear (ZR_1)** - Rotates ZP_0.
18. **Z Position Mechanism Platform (ZR_2)** - The Z Position mechanism sits on this component.
19. **Z Shaft (ZP_0)** - The end-effector is attached to this component. Its rotation is controlled by ZR_1 and position is controlled by ZP_1.
20. **Z Rack (ZP_1)** - Positions ZP_0
21. **Z Position Bracket (ZP_2)** - The rack slides up and down this component.
22. **Z Pinion Bracket (ZP_3)** - Holds the servo motor (Axis 3) in place.
23. **Z Pinion (ZP_4)** - Mounted to servo motor (Axis 3). Moves ZP_1 up and down ZP_2.
24. **Link 2 Shell (SH_0)** - Shell for Link 2, hides wiring and Z-axis mechanism.
25. **Pinion Shell (SH_1)** - Shell that hides servo motor (Axis 2) and its wires.
26. **Cable Sleeve Bracket (SH_2)** - Inserted into SH_0 (interference fit). Supports the cable sleeve.

## 4 Bill of materials summary

The bills of materials are given in Table 3 and 4 for the Manipulator and Motion Controller respectively. Components listed with a ‘Generic’ part number are not tied to a specific brand, are widely available, and can be sourced from any supplier as long as they meet the description. Small hardware items may only be sold in multi-piece packs. The unit prices listed in the BOM reflect the cost per item, not the cost of purchasing an entire pack. As a result, the total cost shown may be lower than the actual amount a user would pay when sourcing materials. All prices reflect supplier listings as of November 2025 and are provided for informational purposes only, as they may vary over time.

**Table 3:** Bill of materials for the Manipulator.

Designator	Description	Component source	Part number	Qty	£/unit	£ total
SER_24	24 kgcm Servo	AGFRC/Amazon	B53DHS	2	16.99	33.98
SER_9G	2.8 kgcm Servo	AGFRC/Amazon	B11DLS	2	5.32	10.64
POT_5K	5k Potentiometer	Amazon/eBay	RV24YN20F B502	2	5.63	11.26
S_M2 × 6	M2 Button Head Hex Screw 6 mm	Amazon/eBay	Generic	2	0.02	0.04
S_M2 × 8	M2 Button Head Hex Screw 8 mm	Amazon/eBay	Generic	17	0.02	0.34
S_M2 × 12	M2 Button Head Hex Screw 12 mm	Amazon/eBay	Generic	17	0.02	0.34
S_M2 × 16	M2 Button Head Hex Screw 16 mm	Amazon/eBay	Generic	7	0.02	0.14
S_M3 × 16	M3 Button Head Hex Screw 16 mm	Amazon/eBay	Generic	2	0.02	0.04
S_M3 × 20	M3 Button Head Hex Screw 20 mm	Amazon/eBay	Generic	7	0.02	0.14
S_M4 × 12	M4 Button Head Hex Screw 12 mm	Amazon/eBay	Generic	8	0.02	0.16
S_M4 × 16	M4 Button Head Hex Screw 16 mm	Amazon/eBay	Generic	6	0.02	0.12
S_M4 × 20	M4 Button Head Hex Screw 20 mm	Amazon/eBay	Generic	4	0.02	0.08
N_M2	M2 Nut	Amazon/eBay	Generic	4	0.02	0.08
WT_1250	Iron Weight Plate 1.25 kg, diameter:	Amazon/eBay	Generic	1	6.99	6.99
	140mm (max), thickness: 20 mm (max)					
JR_EXT	Servo Extension Lead	Amazon/eBay	Generic	10	0.36	3.60
SLV_12	Braided Cable Sleeve 12–20 mm	Amazon/eBay	Generic	0.25 m	2.20/m	0.55
FIL_PLA	PLA Filament	Bambu Lab	PLA Basic	527.97 g	17.99/kg	9.50

					**£ total**	**78.00**

**Table 4:** Bill of materials for the Motion Controller.

Designator	Description	Component source	Part number	Qty	£/unit	£ total
NUC_F4	STM32 Nucleo-64 Development Board	Mouser/RS/DigiKey	NUCLEO-F446RE	1	13.01	13.01
PRO_R3	Prototyping Shield for Arduino Uno R3	Amazon/eBay	Generic	1	1.89	1.89
TER_2P	2-Pin PCB Mount Screw Terminal Block	Amazon/eBay	Generic	1	0.08	0.08
TER_3P	3-Pin PCB Mount Screw Terminal Block	Amazon/eBay	Generic	6	0.08	0.48
CAP_100u	100 uF 50V Through Hole Capacitor	Mouser/RS/DigiKey	Generic	1	0.18	0.18
POW_5V	230V AC to 5V DC (15 W) Adapter	Amazon/eBay	Generic	1	6.99	6.99
DUP_MM	Dupont Wires Male to Male	Amazon/eBay	Generic	20	0.03	0.60
DUP_FM	Dupont Wires Male to Female	Amazon/eBay	Generic	9	0.03	0.27

					**£ total**	**33.50**

## 5 Build instructions

This section provides mechanical and electrical build instructions for the EduSCARA Manipulator and Motion Controller. Recommended tools include:

• Stanley knife for trimming 3D-printed parts.
• Allen key set for tightening bolts.
• Scissors for cutting SLV_12 to length.
• Soldering equipment for attaching components to the PRO_R3 shield.
• Small flathead and Phillips screwdrivers for tightening screws and terminal blocks.

The Manipulator assembly steps are numbered in the top-left corner. The Motion Controller assembly order is flexible, provided all connections match the schematic.

### 5.1 Manipulator build instructions

**Figure:**
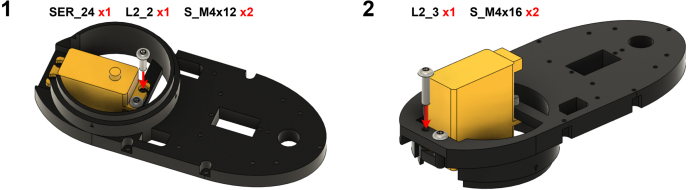

**Figure:**
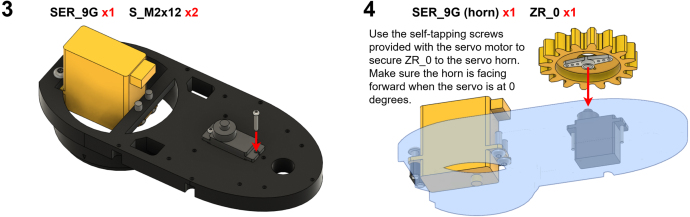

**Figure:**
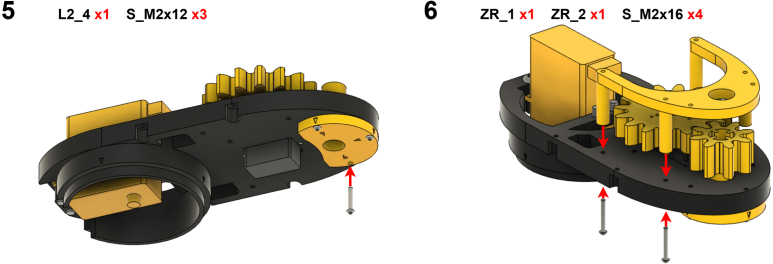

**Figure:**
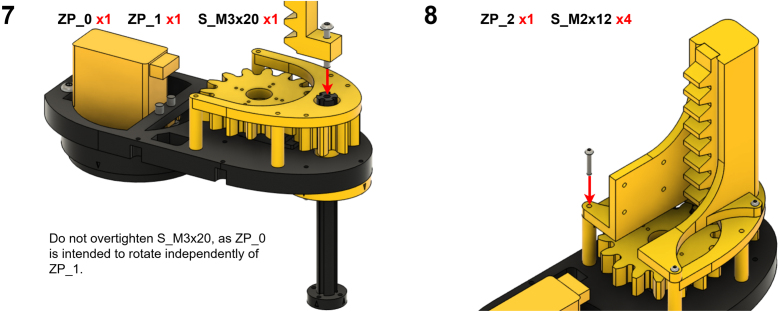

**Figure:**
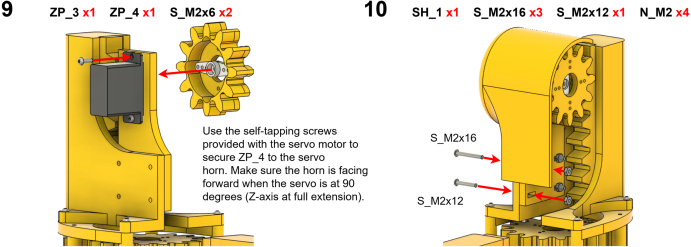

**Figure:**
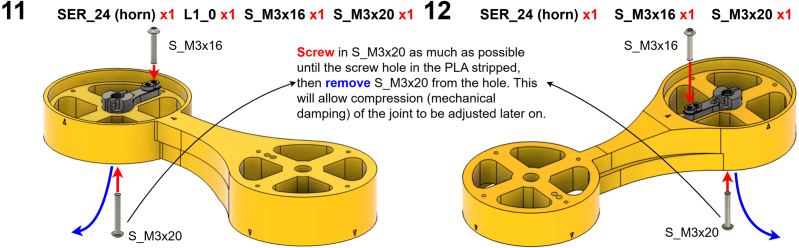

**Figure:**
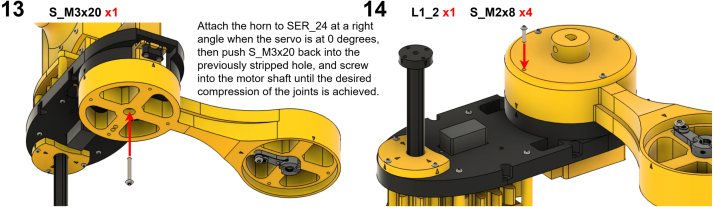

**Figure:**
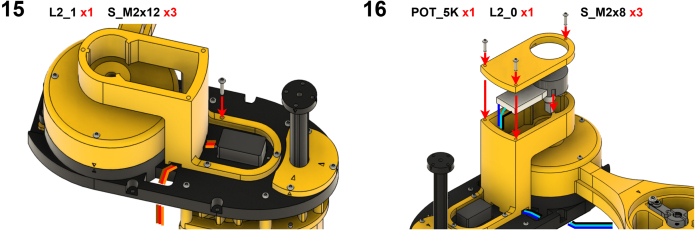

**Figure:**
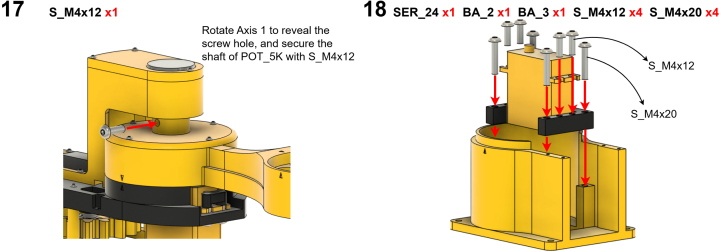

**Figure:**
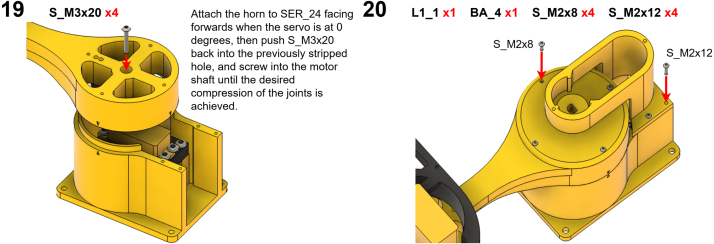

**Figure:**
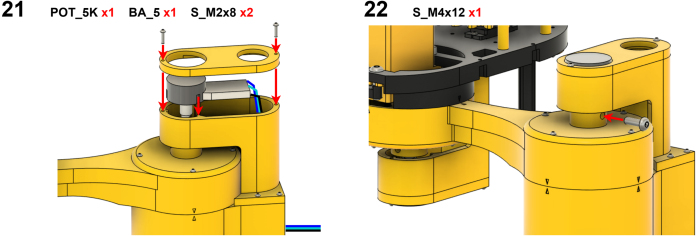

**Figure:**
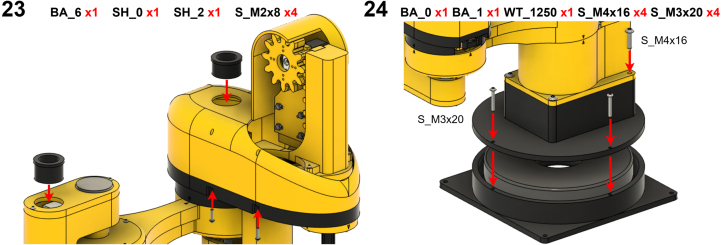

**Figure:**
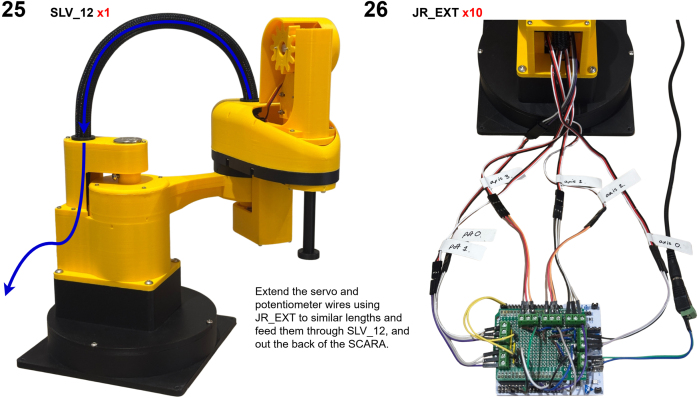

**Fig. 15:**
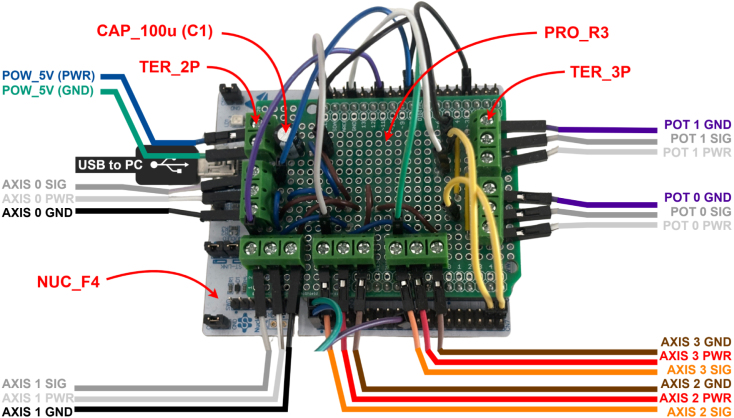
Motion Controller with Connections to Manipulator.

**Fig. 16:**
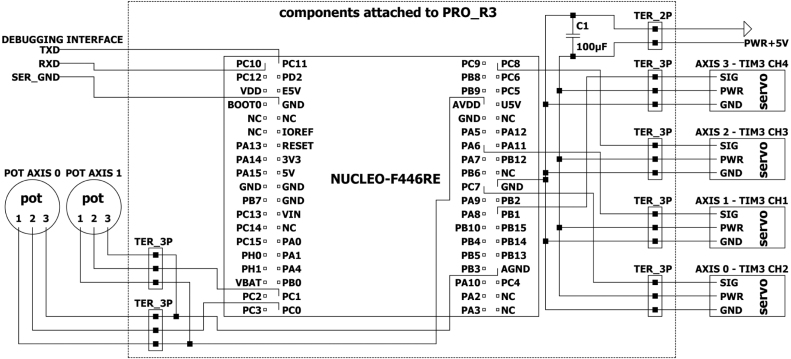
Motion Controller Schematic.

### 5.2 Motion controller assembly guide

Fig. 15 shows the assembled motion controller and Fig. 16 shows its schematic. The motion controller consists of an Arduino shield (PRO_R3) mounted on the NUCLEO-F446RE board (NUC_F4). The shield provides pin terminals (TER_2P x1, TER_3P x6) for connecting the external power supply (POW_5V), the four axes, and the potentiometers. A 15 W power supply (POW_5V) was selected because it provides sufficient current headroom to operate all servos under typical light-load conditions while remaining a low-cost option. The schematic can be used to design a PCB that replaces the Arduino shield in future, along with 3D-printed housing. The motion controller communicates with the PC via the USB port, which also supplies power to the controller logic.

### 5.3 Safety concerns

The authors recommend that users pay particular attention to the following safety scenarios during assembly and operation of the EduSCARA:

• Verify correct polarity for the POW_5V power and ground connections, as reversing them may damage components such as capacitor C1 if a polarised capacitor is used.
• Disconnect power before making or modifying any electrical connections.
• Ensure wires are not trapped between gears or moving parts during assembly.
• When attaching end effectors with cables, such as a motor-driven gripper, provide sufficient cable slack to allow the full operating range without tension or snagging.
• Before running the SCARA_AUTO_CALIBRATE command, raise the Z-axis to avoid collisions, as the calibration routine performs a full-range sweep of Axes 0 and 1.

## 6 Operation instructions

### 6.1 Potentiometer calibration

The accuracy of the EduSCARA is limited by the precision of its position sensors, making proper potentiometer calibration essential. This calibration aligns the potentiometer’s raw ADC values with joint angles, and periodic recalibration may be needed due to sensor drift over time. This procedure is unrelated to the SCARA_AUTO_CALIBRATE API command and PID calibration. The process requires the user to manually move each joint to predefined marker positions, record the corresponding ADC readings, and enter these values into the firmware.

**Fig. 17:**
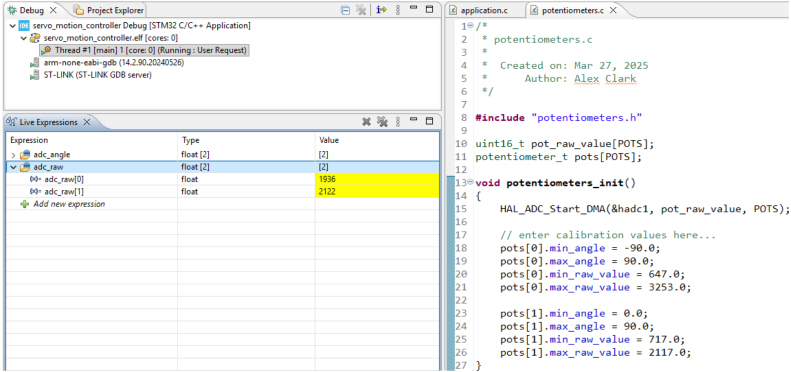
Potentiometer calibration in the firmware.

Open the *servo_motion_controller* project in STM32CubeIDE. The source files of interest are *application.c* and *potentiometers.c*. In *application.c*, comment out the “API” macro, and then start the debugging session. In the Live Expressions tab (see Fig. 17), watch the adc_raw array, and open *potentiometers.c*. Align the calibration markers on each joint and record the corresponding adc_raw readings in the potentiometers_init() function following these steps:

1. Rotate **Axis 0** to −90° and assign adc_raw[0] to pots[0].min_raw_value.
2. Rotate **Axis 0** to 90° and assign adc_raw[0] to pots[0].max_raw_value.
3. Rotate **Axis 1** to 0° and assign adc_raw[1] to pots[1].min_raw_value.
4. Rotate **Axis 1** to 90° and assign adc_raw[1] to pots[1].max_raw_value.

Return to *application.c* and uncomment the “API” macro. Then rebuild and flash the firmware to the microcontroller.

### 6.2 Python API

This section demonstrates how to use the Python API to connect to the Motion Controller and execute initialisation, calibration, motion, and read commands listed in Table 5.

**Fig. 18:**
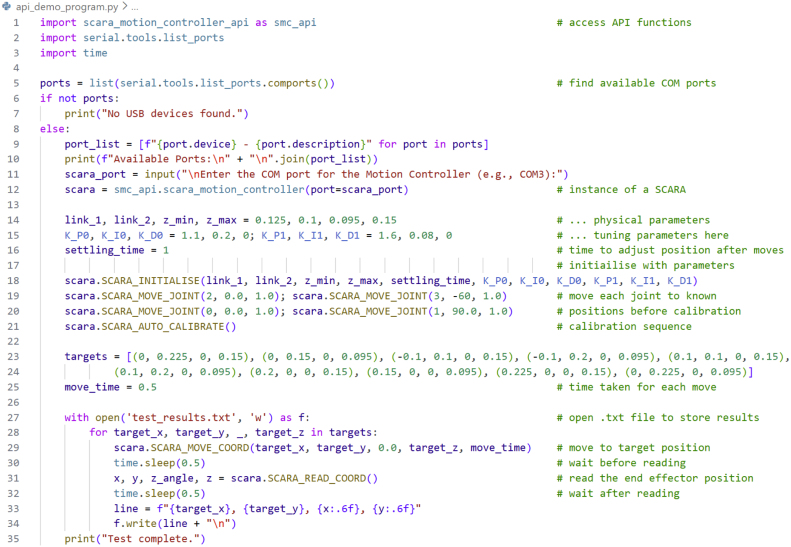
Performance evaluation example program - *api_demo_program.py*.

The example program shown in Fig. 18 can be used to evaluate the performance of the robotic system. This program demonstrates connection, initialisation, auto-calibration, joint-move, cartesian-move, and read commands. Fig. 24 provides a visualisation of the move sequence performed in this program. To run the program, *scara_motion_controller_api.py* must be placed in the same directory.

**Table 5:**
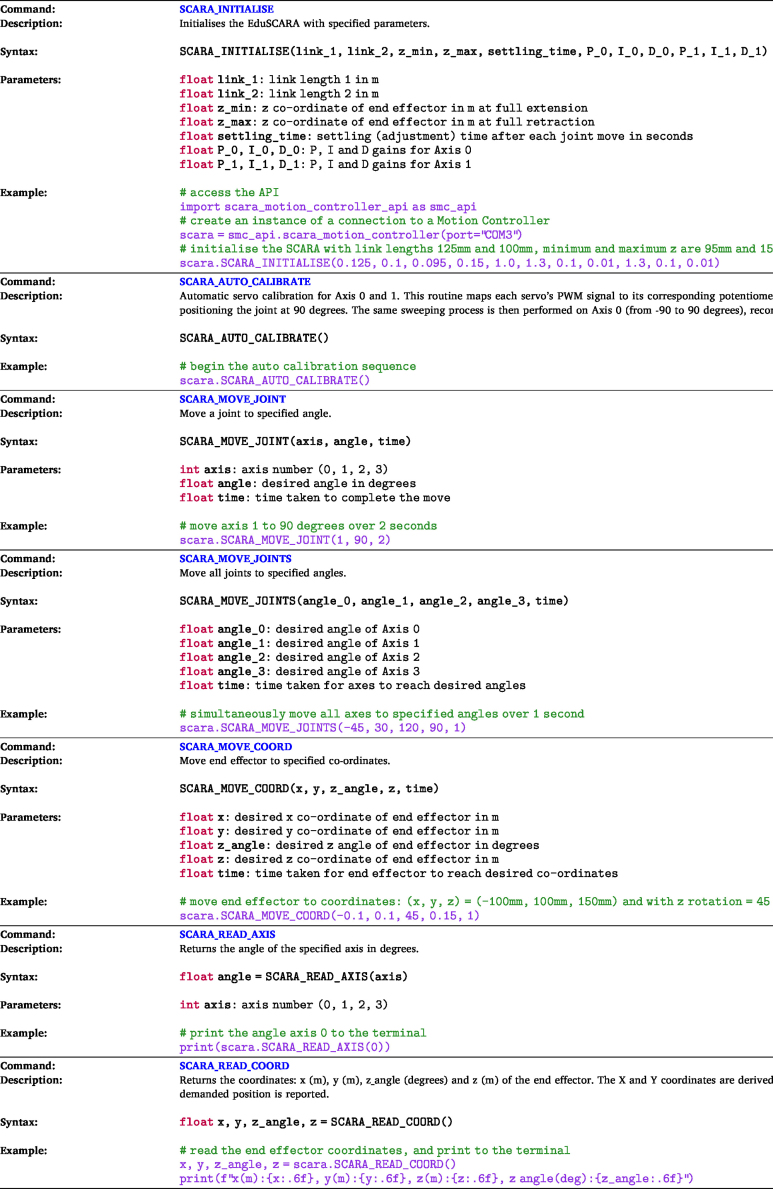
API commands (*scara_motion_controller_api.py*)

## 7 Validation and characterisation

This section demonstrates the EduSCARA’s operation and characterises its performance. It examines the effectiveness of PID tuning, and measures key metrics such as repeatability and payload, which are important for comparison with similar robots in Table 1. The results highlight the system’s strengths and limitations, with a real-world Resistor Sorting application demonstrating both its practical functionality and value as a teaching tool.

### 7.1 Positional repeatability evaluation

**Fig. 19:**
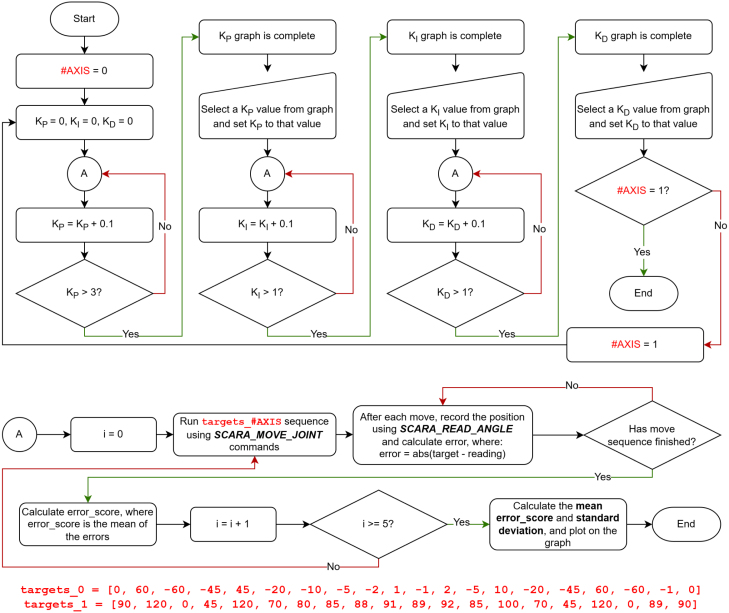
Flow diagram for PID tuning method, using *move_joint_pid_eval.py*.

The positional repeatability of the EduSCARA was evaluated using fast 0.5 s moves with no end-effector payload. X–Y repeatability is assessed using feedback available on axes 0 and 1, accessed through the API’s position read commands. Although the low-cost potentiometers chosen have a nominal 10% tolerance and mechanical travel variation, these effects are compensated for through a per-joint calibration procedure that maps each potentiometer’s output to its true angular position, as detailed in the Potentiometer Calibration section. Consequently, measurement accuracy depends less on the potentiometer tolerance and more on the quality of the user’s calibration. The Z-rotation and position axes (2 and 3) operate in open loop and cannot be tuned in software; however, their repeatability was characterised manually. As described in Fig. 9, axis 3 uses a rack-and-pinion mechanism, which showed <0.1mm total variation over 10 repeated up–down cycles when measured using a Vernier caliper, and axis 2 uses a printed 1:2 gear-ratio mechanism, which was found exhibit approximately 3 degrees of backlash. X–Y repeatability evaluation begins by identifying the PID gains for Axes 0 and 1 that produce the minimum joint angle error. This tuning process is performed using the *move_joint_pid_eval.py* script, outlined in Fig. 19.

**Fig. 20:**
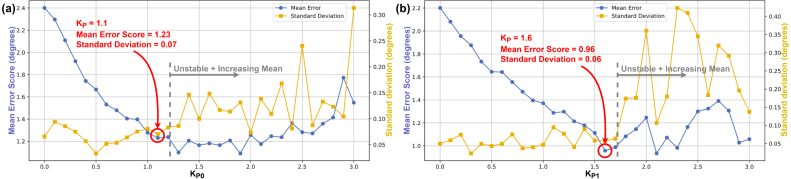
Mean Error Score and Standard Deviation vs $K_{P}$ for Axis 0 ($K_{I0}=0$, $K_{D0}=0$) (a) and Axis 1 ($K_{I1}=0$, $K_{D1}=0$) (b).

**Fig. 21:**
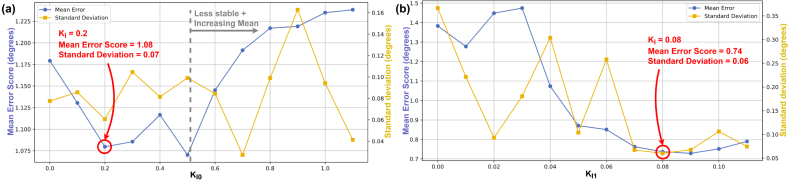
Mean Error Score and Standard Deviation vs $K_{I}$ for Axis 0 ($K_{P0}=1.1$, $K_{D0}=0$) (a) and Axis 1 ($K_{P1}=1.6$, $K_{D1}=0$) (b).

**Fig. 22:**
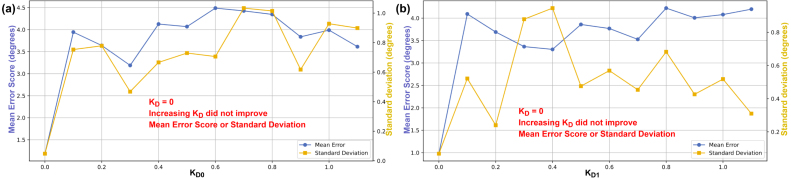
Mean Error Score and Standard Deviation vs $K_{D}$ for Axis 0 ($K_{P0}=1.1$, $K_{I0}=0.2$) (a) and Axis 1 ($K_{P1}=1.6$, $K_{I1}=0.08$) (b).

The selected PID gains were $K_{P0}=1.1,K_{I0}=0.2,K_{D0}=0$ for Axis 0, and $K_{P1}=1.6,K_{I1}=0.08,K_{D1}=0$ for Axis 1. Annotated results from the tuning procedure are shown in Fig. 20, Fig. 21, Fig. 22.

Increasing $K_{P}$ initially reduces the mean error score, but beyond a certain point the system becomes unstable, indicated by rising and irregular mean error scores and standard deviations. The chosen proportional gains, $K_{P0}=1.1$ and $K_{P1}=1.6$, correspond to the minimum stable mean error score for each axis.

The effect of increasing $K_{I0}$ was less obvious than that of $K_{P0}$. The value of $K_{I0}=0.2$ was selected because it reduced the mean error score relative to $K_{I0}=0$ without increasing the standard deviation. For Axis 1, an initial $K_{I1}$ sweep from 0 to 1 in 0.1 increments produced no meaningful trend. Reducing the range to 0 to 0.1 with 0.01 increments revealed a clear mean error score improvement up to $K_{I1}=0.08$, which was the selected value.

**Fig. 23:**
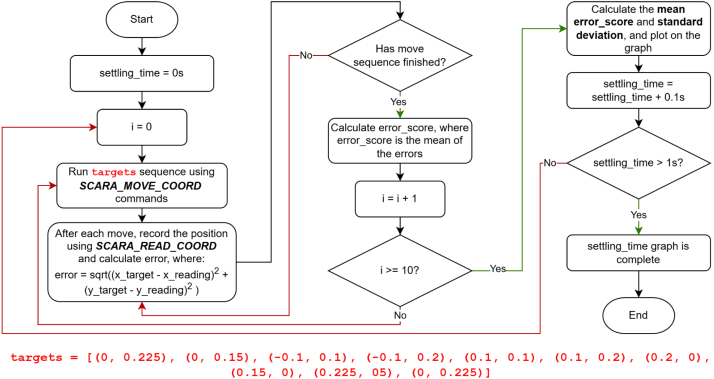
Flow diagram for testing X-Y repeatability and settling_time, using *move_coord_settling_time_eval.py*.

Increasing $K_{D}$ degraded performance for both axes and was therefore set to 0. This is likely due to noise in the potentiometer measurements, as the derivative term amplifies high-frequency noise because it responds to rapid changes in the signal. As a result, even small measurement fluctuations produce large, erratic control outputs, leading to poor stability.

**Fig. 24:**
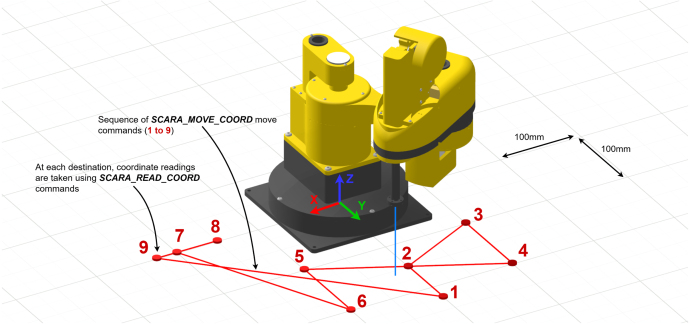
Visualisation of the move sequence used to evaluate X-Y repeatability.

With the PID gains selected, X-Y repeatability was evaluated using the *move_coord_settling_time_eval.py* script, which also tests the effect of adjusting settling_time. The method and target sequence are shown in Fig. 23, Fig. 24.

**Fig. 25:**
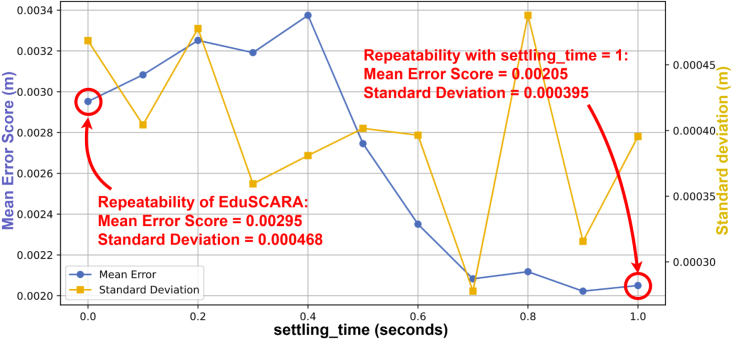
Mean Error Score and Standard Deviation vs Settling Time.

The results obtained from 0.5 s moves with settling_time = 0 are used to calculate the rated repeatability of the EduSCARA, as settling_time is optional and independent of the PID gains. Annotated results are presented in Fig. 25.

Increasing settling_time improves X-Y repeatability by allowing additional time for the end-effector to converge to its final position. Using the baseline results (settling_time = 0), the system achieved a mean error score of 0.00295 m with a standard deviation of 0.000468 m. Defining repeatability as the mean error score plus one standard deviation and rounding up to the nearest 0.1 mm yields a repeatability rating of **± 3.5 mm** for the chosen PID parameters. The complete set of test data to obtain this result is accessible in the project repository.

### 7.2 Payload evaluation

The absolute maximum payload of the EduSCARA is limited by the rack-and-pinion Z-axis (Axis 3). A force test using a Newton meter showed that approximately 12N (1.2 kg) is required to pull the Z-axis downwards while the servo was engaged — a value far beyond what the arm structure can safely support. To determine a realistic rating, the test was repeated by applying force only until noticeable flex occurred in the arm. This threshold was approximately 0.2N (0.02 kg). Halving this value gives the rated payload of **100g** for the EduSCARA.

**Fig. 26:**
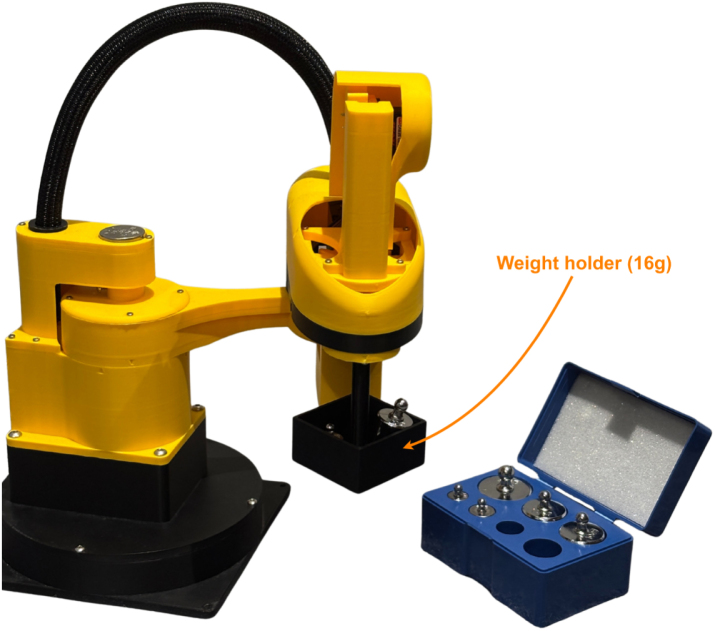
Payload testing setup, showing EduSCARA with weight holder attachment.

**Fig. 27:**
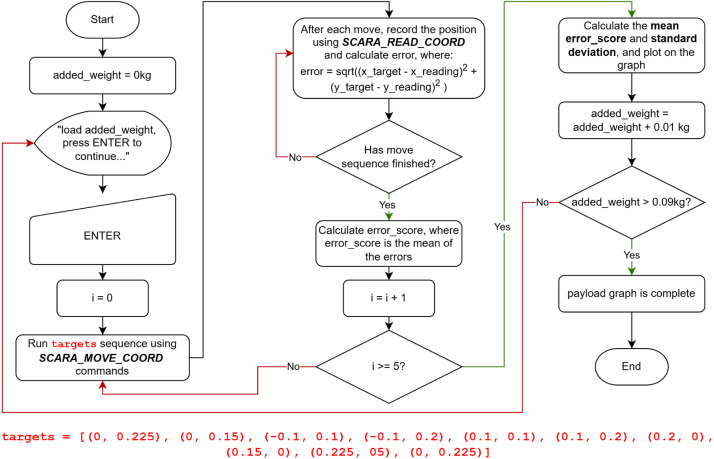
Flow diagram for testing X-Y repeatability with increasing payload, using *move_coord_payload_eval.py*.

**Fig. 28:**
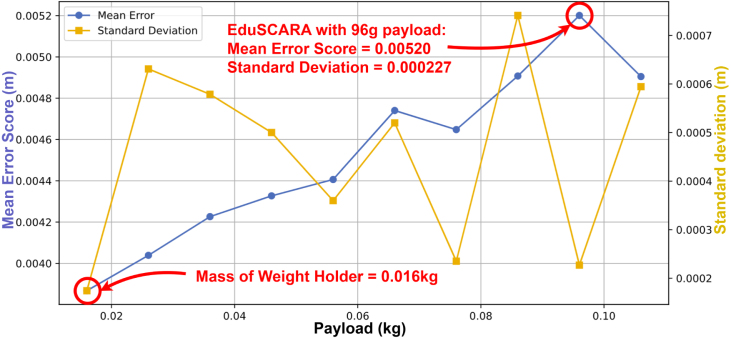
Mean Error Score and Standard Deviation vs Payload.

The effects of increasing payload on X-Y repeatability were evaluated using the same test conditions as the baseline assessment using the *move_coord_payload_performance.py* script (0.5 s moves, settling_time = 0). The payload setup is shown in Fig. 26, and the procedure is illustrated in Fig. 27. Annotated results are presented in Fig. 28.

As expected, X-Y repeatability decreases with increasing payload. For the rated payload, using the mean error score (0.00520 m) plus one standard deviation (0.000227 m) and rounding up to the nearest 0.1 mm gives a repeatability rating of **± 5.5 mm**. This value could likely be improved by re-tuning the PID parameters while operating under the rated payload.

### 7.3 Teaching exercise example – Resistor sorter

Difficulty in identifying resistors in university labs often lead to the unnecessary disposal of functional components. The proposed teaching exercise uses the EduSCARA platform to address this issue, while demonstrating its capabilities both as an effective educational tool and a useful robotic system through the development of an automated through-hole resistor sorter, providing students with a practical opportunity to apply robotics skills in a real-world context.

**Fig. 29:**
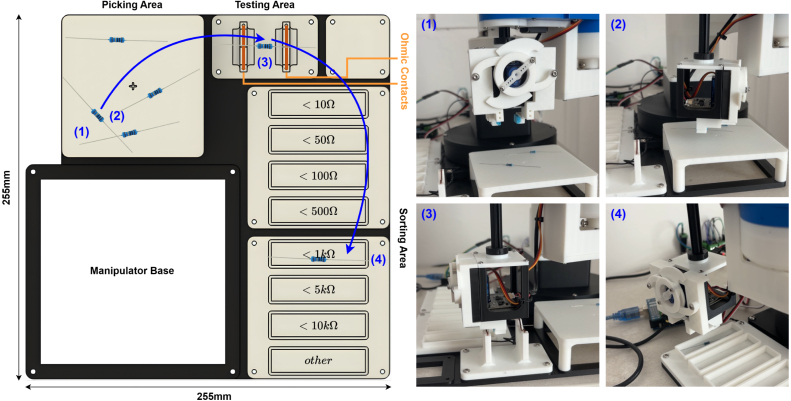
Automated through-hole resistor sorting rig and process.

This exercise aims to integrate the EduSCARA with a custom end-effector and a camera to locate (1), pick up (2), test (3), and sort (4) resistors based on their resistance values. The rig for this exercise is provided as a 3D-printable CAD model, and the sorting process within this setup is illustrated in Fig. 29.

**Fig. 30:**
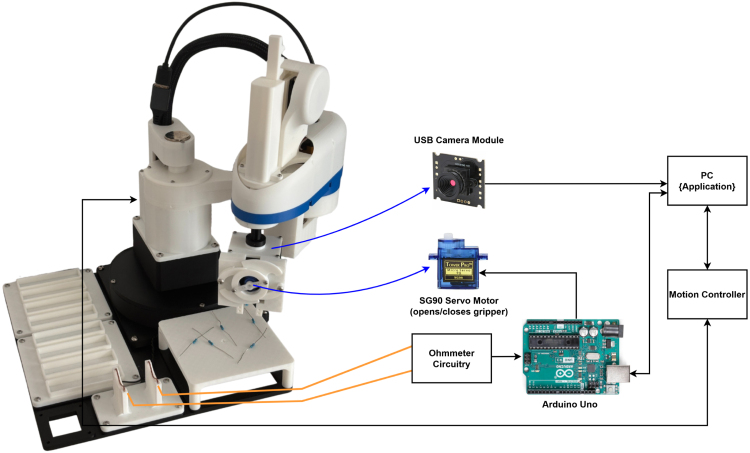
Automated through-hole resistor sorter system.

While not the only possible solution, Fig. 30 presents a successful approach to the task and serves as a reference for both implementing a working system and understanding the learning opportunities. This example uses a parallel gripper mechanism as the end effector, which also houses a downward-facing USB camera module. When the gripper opens, the camera gains a clear view between the claws. Due to the limited field of view and height constraints, the manipulator ensures visual coverage by moving to predefined positions around the tray. An Arduino is used to control both the gripper and ohmmeter circuitry. It contains firmware that receives commands from the PC to either open or close the gripper or take an ohmmeter reading.

**Fig. 31:**
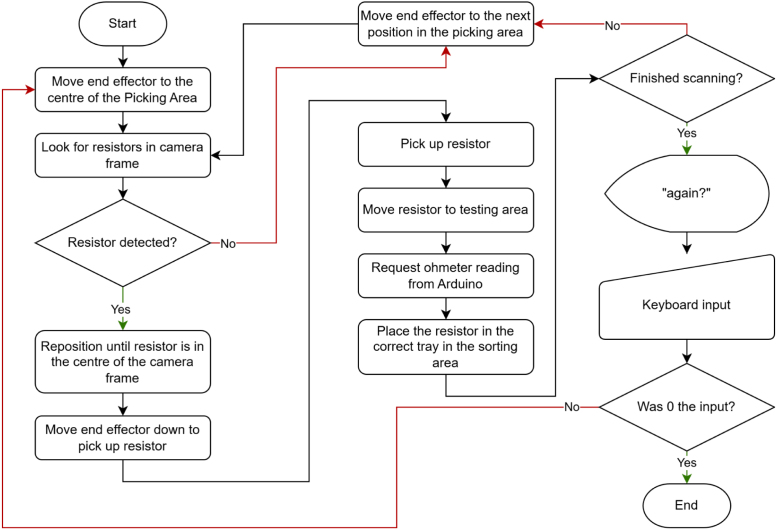
Automated through-hole resistor sorter flow diagram.

The Python application running on the PC handles both image processing and motion control. A resistor detection algorithm based on the YOLO (You Only Look Once) framework [29] is used to identify resistors within the camera’s field of view. YOLO identifies the object it sees is a resistor and detects its position and orientation; it does not guess its resistance value. Once a resistor is detected, the manipulator iteratively adjusts the end-effector position until the resistor is centred in the frame, aligning it with the gripper claws enabling it to be picked up. The completed resistor sorting application is described in Fig. 31.

Through completing this project, students apply and develop a wide range of practical engineering skills, including:

1. Computer-aided design (CAD) and 3D printing for the end-effector.
2. Embedded systems development for the Arduino and related circuitry.
3. Machine learning model training and integration for the resistor detector.
4. Path planning and system integration, dealing with and compensating for real-world non-idealities, such as PID tuning on the joints to minimise vibration on the camera while maximising speed of the manipulator motion.

This exercise is well-suited for both individual and group work, depending on the desired learning outcomes. When completed individually, students gain end-to-end experience in robotics development. In a group setting, the exercise promotes collaboration and project management skills, allowing team members to focus on specific subsystems aligned with their interests and strengths, supporting deeper skill development in particular aspects of the project.

### 7.4 Capabilities and limitations

The EduSCARA platform is designed as a low-cost educational SCARA that balances accessibility with industrially relevant motion-control concepts. The following points summarise its key capabilities and current technical limitations.

**Capabilities**

• The EduSCARA is rated for a 100 g payload and achieves ± 3.5 mm repeatability under no-load conditions using fast 0.5s moves without post-move angle correction, providing sufficient precision and capacity for educational demonstrations of kinematics and closed-loop control as well as small-scale applications such as the Resistor Sorting example.
• Customisable 3D-printed manipulator implements an industrially relevant RRPR SCARA configuration using widely available, low-cost components.
• Axes 0 and 1 implement PID-tunable closed-loop control, with an optional post-move joint-angle correction routine to improve final positioning accuracy.
• All moves are executed using a double-S motion profile, reducing jerk and providing smoother acceleration and deceleration characteristics.
• Python API supports initialisation, automatic calibration, motion, and communication commands, while also enabling integration with machine learning and computer vision applications, as demonstrated in the Resistor Sorting example.

**Limitations**

• The use of hobby-grade servo motors keeps the system lightweight, inexpensive, and easy to control, but their deadband and internal-gear backlash introduce lost motion and prevent the level of precision achievable with stepper motors.
• Position feedback on Axes 0 and 1 are provided by potentiometers, which are simple to integrate but introduce noise and rely on careful user calibration, limiting the accuracy and stability.
• The 3D-printed links constrain the system’s payload capacity, and the current design requires structural reinforcement to safely handle heavier loads.
• The Motion Controller does not support on-device programmability, and the current API offers only a small set of motion commands, limiting the system’s ability to perform advanced path-planning operations.

### 7.5 Recommendations for future work

The EduSCARA platform meets its primary goal of providing a low-cost, functional, and educational robotics system, but several limitations remain. Many of these can be reduced by choosing alternative hardware.

Although the choice to use servo motors was justified, from a performance standpoint, stepper motors would offer higher precision and eliminate backlash due to their gearless design. However, their increased weight would likely require structural reinforcement, such as metal rods within the links. Potentiometers, while simple to integrate, introduce noise that limits accuracy and stability. Incremental encoders provide cleaner signals but require homing hardware and routines, while absolute encoders remain too costly. Stepper motors with integrated encoders are a strong choice, however, the high precision open-loop performance of the stepper motors may obscure the effects of control algorithms, making it harder for learners to observe and understand system behaviour and therefore hurting its educational value, though it would enable the platform to support more advanced high accuracy applications.

Further software developments could also strengthen the platform’s industrial relevance. Enabling on-device programmability within the Motion Controller would bring it closer to an industrial PLC, though it would require a more complex firmware architecture, likely built on an RTOS. Additional enhancements such as broader API language support, ROS integration, and more advanced motion commands for complex path planning would further extend the system’s capabilities.

Overall, both the hardware and software offer clear opportunities for improvement that would make EduSCARA more capable and more reflective of real industrial systems. Continued development in these areas is encouraged and would help the platform evolve into a more complete and versatile tool for hands-on robotics education.

## CRediT authorship contribution statement

**Alex Clark:** Writing – original draft, Validation, Software, Project administration, Methodology, Investigation, Formal analysis, Conceptualization. **Uriel Martinez-Hernandez:** Writing – review & editing. **Tareq Assaf:** Writing – review & editing, Supervision, Project administration, Conceptualization.

## Ethics statements

This work did not involve human subjects or animal experiments.

## Declaration of competing interest

The authors declare that they have no known competing financial interests or personal relationships that could have appeared to influence the work reported in this paper.
